# Mutational Landscapes of Sequential Prostate Metastases and Matched Patient Derived Xenografts during Enzalutamide Therapy

**DOI:** 10.1371/journal.pone.0145176

**Published:** 2015-12-22

**Authors:** Manish Kohli, Liguo Wang, Fang Xie, Hugues Sicotte, Ping Yin, Scott M. Dehm, Steven N. Hart, Peter T. Vedell, Poulami Barman, Rui Qin, Douglas W. Mahoney, Rachel E. Carlson, Jeanette E. Eckel-Passow, Thomas D. Atwell, Patrick W. Eiken, Brendan P. McMenomy, Eric D. Wieben, Gautam Jha, Rafael E. Jimenez, Richard Weinshilboum, Liewei Wang

**Affiliations:** 1 Department of Oncology, Mayo Clinic, Rochester, Minnesota, United States of America; 2 Division of Biomedical Statistics and Informatics, Department of Health Sciences, Mayo Clinic, Rochester, Minnesota, United States of America; 3 Masonic Cancer Center and Department of Laboratory Medicine and Pathology, University of Minnesota, Minneapolis, Minnesota, United States of America; 4 Department of Radiology, Mayo Clinic, Rochester, Minnesota, United States of America; 5 Department of Biochemistry and Molecular Biology, Mayo Clinic, Rochester, Minnesota, United States of America; 6 Division of Hematology-Oncology, University of Minnesota, Minneapolis, Minnesota, United States of America; 7 Department of Pathology and Lab Medicine, Mayo Clinic, Rochester, Minnesota, United States of America; 8 Department of Molecular Pharmacology and Experimental Therapeutics, Mayo Clinic, Rochester, Minnesota, United States of America; University of Kentucky College of Medicine, UNITED STATES

## Abstract

Developing patient derived models from individual tumors that capture the biological heterogeneity and mutation landscape in advanced prostate cancer is challenging, but essential for understanding tumor progression and delivery of personalized therapy in metastatic castrate resistant prostate cancer stage. To demonstrate the feasibility of developing patient derived xenograft models in this stage, we present a case study wherein xenografts were derived from cancer metastases in a patient progressing on androgen deprivation therapy and prior to initiating pre-chemotherapy enzalutamide treatment. Tissue biopsies from a metastatic rib lesion were obtained for sequencing before and after initiating enzalutamide treatment over a twelve-week period and also implanted subcutaneously as well as under the renal capsule in immuno-deficient mice. The genome and transcriptome landscapes of xenografts and the original patient tumor tissues were compared by performing whole exome and transcriptome sequencing of the metastatic tumor tissues and the xenografts at both time points. After comparing the somatic mutations, copy number variations, gene fusions and gene expression we found that the patient’s genomic and transcriptomic alterations were preserved in the patient derived xenografts with high fidelity. These xenograft models provide an opportunity for predicting efficacy of existing and potentially novel drugs that is based on individual metastatic tumor expression signature and molecular pharmacology for delivery of precision medicine.

## Introduction

Intra-tumoral heterogeneity may underlie hitherto unknown adaptive mechanisms of resistance to drug therapy [1]. Recent advances in massively parallel next-generation sequencing have made it possible to study cancer heterogeneity and clonal evolution within cancer [2]. This has led to in-depth elucidation of tumor specific genetic profiles of several cancers including aggressive and lethal prostate cancer [3,4,5]. However, identification of genes that are somatically mutated or have an altered expression profile has yet to yield clinical benefit for patients with advanced prostate cancer. One reason for this could be that functional and therapeutic understanding of these genomic and transcriptomic observations is lacking. Patient derived Xenografts (PDXs) can transform genomic profiles into pharmacogenetic platforms for drug testing [6,7] and offer the potential for enhancing gains from sequencing studies. However, PDX models of prostate cancer have historically been challenging to establish, with a high degree of variability in take-rates reported [8]. Perhaps not surprisingly, the take-rate with metastatic prostate cancer appears to be generally higher than the take-rate with primary prostate cancer [9]. This signifies that PDX models developed from biopsies of advanced prostate cancer may represent feasible platforms for elucidating mechanisms of response to the newest generations of therapies [10,11].

In this study, in a single patient we initially describe and then compare the mutational landscapes of metastatic tissue with PDXs from bone tissue metastasis of a castrate-resistant prostate cancer patient before and after initiating treatment with enzalutamide, a second-generation AR antagonist. We also determine the recapitulation of genomic alterations from serially obtained cancer tissue in animal models.

## Material and Methods

### Clinical Case Presentation

The case presented was part of a prospectively conducted feasibility study performed in February 2013 approved by the Mayo Clinic Institutional Review Board. The goal of the study was to develop PDX models in immune-deficient mouse models from metastases of castration resistant prostate cancer (CRPC) stage patients undergoing standard of care stage specific therapy. PDX models were generated only in one of nine enrolled patients which is presented in this case report. Written informed consent was given by all participants for their clinical records to be used in this study including the patient with successful generation of the PDX models before and after enzalutamide therapy. The patient initially presented with recurrent urinary obstructive symptoms in June 2010. An investigative workup revealed a hard and enlarged prostate and transurethral resection demonstrated an adenocarcinoma with an initial Gleason score (GS) of 9 and a presenting (June 2010) prostate specific antigen (PSA) level of 0.95 ng/dL. He underwent nerve-sparing radical resection of the prostate on October, 2010, with final pathology revealing GS 9; pT3b (bilateral seminal vesicles involvement); N0 (0 of 20 lymph nodes positive for metastases) stage and several positive margins. He received adjuvant radiation combined with luteinizing hormone releasing hormone (LHRH) analogues which were continued for a year. Post therapy his PSA remained undetectable until August 2012 when it increased to 2.9 ng/dL. Staging bone and computed tomography (CT) scan of the abdomen and pelvis were non-revealing for metastasis, but an acetyl choline positron-emission tomography (PET)-CT demonstrated multiple sites of bony metastasis. He was re-started on leuprolide acetate in October 2012, with bicalutamide for hormone sensitive stage and after an initial response progressed to CRPC stage in February, 2013. Prior to initiating therapy for CRPC stage disease, he enrolled on the feasibility study and underwent a research study biopsy of a metastatic rib lesion for sequencing and developing PDX models. He was treated with enzalutamide in combination with sipuleucel-T and indoximod on a clinical trial and underwent a second research biopsy after 12 weeks of continuous treatment with the above combination. At 12 weeks his serum PSA was observed to be 29.3 ng/ml (serially increased from pre enzalutamide PSA of 17.7 ng/ml after two measurements six weeks apart). His repeat imaging scan compared to baseline demonstrated progressive disease in the bone metastasis on a bone scan resulting in change of therapy to systemic docetaxel chemotherapy for 4.5 months and subsequently to carboplatin based chemotherapy after further progression on docetaxel.

### Patient Derived Xenograft Methods

PDXs were developed from biopsies (10mm height x 1.5mm diameter) of castrate-resistant prostate cancer (CRPC) tissue metastases collected from the patient before and after initiating stage specific standard of care treatment with enzalutamide. Renal capsule xenografting and subcutaneous 25mg testosterone pellet (Jungle Jim, Fairfield, OH) implantation in NOD-SCID mice (Jackson laboratories, Bar Harbor, Maine; Charles River laboratories, Raleigh, North Carolina) were performed as described previously for the evaluation of prostate carcinogenesis and benign prostatic hyperplasia [12]. Expansion of sub-renal xenografts was observed 4 and 6 months after implantation of baseline (visit 1) and visit 2 (after 12-weeks of continuous enzalutamide therapy) biopsy tissue, respectively. PDX tumors from each visit were further expanded in second-generation mice for molecular characterization. (Details of mice anesthesia and euthanasia are included in **S1 Animal Methods**.) The initial xenograft mice were not biopsied again. The mice were sacrificed when their xenograft tumor diameter achieved 1.0~1.5cm, at which point the whole tumors were harvested. A portion of tumors were re-injected into the second generation mice. The rest of tumors were collected for molecular analyses, pathology confirmation or frozen for future use. Whole exome (WES) and transcriptome sequencing (RNA-seq) were performed on patient tumor tissues and on 10 PDXs (four models from the first generation and six models from the second generation) (**S1 Fig**).

### Whole Exome Sequencing (WES) of tumor

Deep sequencing of exome captured DNA was performed on the Illumina HiSeq 2500. Exome capture was performed using a customized version of Agilent’s SureSelect Human All Exon V4+UTR capture kit with additional baits added to cover exons and introns of the androgen receptor (AR) gene [13]. DNA samples were captured following the manufacturer’s protocol and sequenced as 101 nt pair-end reads in a single lane of the HiSeq 2500.

We used Genome_GPS, a comprehensive secondary analysis pipeline for high-throughput genome sequencing data developed by Mayo Clinic, to perform sequence alignment, variants calling including single nucleotide variations (SNVs), small insertions and deletions (INDELs), structural variations, and annotation. Unless otherwise specified, all tools built into Genome_GPS were run under the default configurations. Specifically, raw reads were aligned to the human reference genome (hg19/GRCh37) using Novoalign (VN:V2.07.13) with the following options:—hdrhd off -v 120 -c 4 -i PE 425,80 -x 5 -r Random (http://www.novocraft.com/). Realignment and recalibration were performed using GATK [14](VN: 2.7-4-g6f46d11) Best Practices version 3. Germline variations were called with GATK’s UnifiedGenotyper and SNVMix2[15]. Variant quality score was recalibrated using the following command-line optimizations: for SNVs, -an QD -an MQRankSum -an ReadPosRankSum -an FS -an DP; and for INDELs, -an DP -an FS -an ReadPosRankSum -an MQRankSum—maxGaussians 4. Somatic SNVs were called using SomaticSniper (VN: 1.0.0.1–19) [16], JointSNVMix2 (0.8b2)[17] with the “-prob 0.1” parameter, or MuTect (1.1.4), and Somatic INDELs were called using SomaticIndelDetector (GATK) and annotated with SnpEFF (VN: 3.0c). Reported SNVs and INDELs were filtered to exclude any variant found in the normal blood sample, variants outside the exons, variants of low or modifier impact, and any variant observed in the 1000 Genomes project [18] or the Exome Sequencing Project [19].

All somatic copy number variations (CNVs) were called using an updated version of PatternCNV [20], which uses reference samples to learn the pattern and variance of the WES coverage to better enable somatic CNV calling. The median reads per kilobase per million mapped reads (RPKM) coverage and variance of a set of germline samples was computed for each 120bp interval overlapping with the location of the exome capture probes. Probes GC content bias was corrected by grouping the probes according to their GC content and by the number of copies of each probe in the capture pool. The peak density within each group was then adjusted on a per sample basis. The base-2 logarithm ratio (Log_2_R) of the coverage between a tumor sample and the reference set was computed for each probe. The Log_2_R values were segmented using CBS [21] in the DNAcopy package, taking the predicted variance into account (adjusted for the coverage of the tumor sample). The log_2_R was then normalized so that the median of all segmented probe values = 0. After manual review and cross-sample comparison, a few samples were re-centered so that chromosome 2 had the same copy number. A threshold of |Log_2_R| > 0.5 was used to call a region as amplified or deleted. Tumor purity was estimated using PurBayes, with some modifications (1). Specifically, we modified PurBayes to simultaneously identify subclones and estimate purity for each subclone across multiple sequential time points. The criteria to estimate purity included limitation to variants that had at least 50X coverage, did not overlap a called CNV (|Log_2_R| ≥ 0.5) and a dbSNP variant, was not on chromosome M or chromosome X, and had at most one read carrying the mutant allele in the corresponding germline sample. Lastly, we eliminated variants that could have arisen from reads mapping to multiple locations by taking a sequence region of 101bp region centered on the variant and aligning against the human genome (hg19) using BLAT [22]. Any variant with multiple genome mappings by this approach was not used in the purity analysis.

### RNA sequencing (RNAseq) of tumor specimens

mRNA libraries were prepared using Illumina’s TruSeq RNA prep kit and standard protocol. The RNA libraries were sequenced as 101 nt pair-end reads at 2 samples per lane of an Illumina HiSeq 2500, generating an average of 140 million reads per sample.

Mayo Analysis Pipeline for RNA-Seq (MAP-RSeq) is the comprehensive secondary analysis pipeline for RNAseq data at Mayo Clinic [23]. The MAP-RSeq workflow performs a thorough processing of paired-end RNAseq data in three steps which included alignment and parameter optimization; quality control of samples and summary of genomic features per sample or run. These three steps were run using the following options:

1. Alignment and parameter optimization: Reads were aligned by TopHat 2.0.6 [24] using the hg19 genome build and bowtie1 aligner option [25]. TopHat requires mean and standard deviation of fragment size to perform alignment. Hence, fragment size parameters were estimated by sampling the first 100K read pairs that were uniquely mapped. Most of the tools in MAP-RSeq use the default configurations; however, we used the following settings for TopHat:—keep-fasta-order,—keep-tmp,—no-coverage-search, —bowtie1,—library-type fr-unstranded,—max-multihits 20,—solexa1.3-quals,—fusion-search,—fusion-ignore-chromosomes chrM,—fusion-min-dist 50000.
2. Quality control of samples: MAP-RSeq also provides detailed quality-control plots per sample to estimate the distance between paired-end reads, evaluate the sequencing depth for alternate splicing analysis, determine the rate of duplicate reads, and evaluate coverage of reads across genes using RSeQC software (http://rseqc.sourceforge.net/) [26].
3. Summary of genomic features per sample or run: Gene expression counts were generated using HTseq software (http://www-huber.embl.de/users/anders/HTSeq/doc/overview.html) from Illumina gene annotation files (http://support.illumina.com/sequencing/sequencing_software/igenome.html). The MAP-RSeq also provides a list of expressed fusion transcripts using the TopHat-Fusion algorithm [27].

### Xenograft sequencing Methods

Sequencing data from patient-derived xenograft (PDX) samples contain a mixture of reads originating from the host (mouse) and from the graft (human). Since the proportion of reads from the different sources can vary significantly across PDXs, classifying reads by source is useful to improve the precision of analysis. For both DNA and RNA, we employed the classification scheme proposed by Conway *et al*. [28], which classifies reads as “graft”, “host”, “both”, “neither” or “ambiguous”. We used the Non-Obese Diabetic (NOD) genome sequences as the host reference genome (http://cgd.jax.org/tools/Seqnature.shtml), where NOD is the background strain of the SCID strain, *NOD*.*CB17-Prkdcscid/J*, used in the engrafting. Using this host reference genome rather than the mouse reference genome (mm10) allowed for less reads to be categorized as “ambiguous” and more being categorized as “host”. For both RNA and DNA, we chose to exclude only those classified as “host” to maximize the amount of graft-originating reads for downstream analysis. Then, in each case, we proceeded with the relevant workflow (Genome_GPS for WES and MAPR-seq for RNAseq) as described previously.

### Recall and Precision

We use recall (i.e. sensitivity) and precision metrics to measure the relevance between tumor and xenograft genomes:

$$recall=\frac{P\cap X}{P}$$

$$precision=\frac{P\cap X}{X}$$

Where *P* denotes the “mutations called from patient tissues” and *X* denotes the “mutations called from xenograft”.

Somatic mutations selected for this analysis had to be called in either the patient tumors or xenografts, had to have high or moderate impact under SNPEff, had to not be found in the 1000 genome European or African samples, had to have 0 reads in the germline, and the 100-bp region surrounding the variant was required to map uniquely to the human reference genome (≥ 90% identity).

### AR variant 7 and AR full length expression

*AR* variant 7 (*AR-V7*) and *AR* full length (*AR-FL*) are the two commonly expressed isoforms in castrate resistant stage. Since *AR-V7* and *AR-FL* share the first 3 exons, expression level of *AR-V7* was therefore measured by the number of splice reads that span exon-3 (chrX:66905852–66905968) and the downstream cryptic exon (chrX:66914515–66915580) located in intron-3. The expression levels of *AR-FL* were measured by the number of splice reads that span exon-4 (chrX:66931244–66931531) and exon-5 (chrX:66937320–66937464). The number of splice reads were then normalized by total splice reads to correct sequencing depth (i.e. SRPM, splice read per million).

### Quantitative real time RT-PCR validation

RNA samples were extracted from snap-frozen xenograft tumor tissues by miRNeasy mini kit (Qiagen, Redwood city, CA). QRT-PCR primers were designed and synthesized to cover the possible regions of gene fusion breakpoints. The GAPDH primer was purchased from Qiagen. The QRT-PCR was performed using the one-step QRT-PCR kit (Qiagen).The amplification Ct value was normalized to that of GAPDH. The PCR amplified products were visualized on agarose gels.

## Results

### Sequencing statistics

A total of 13 metastatic tissue and PDX tumor specimens were obtained for whole exome sequencing (WES) which included two patient tumor tissue specimens (visit 1 and visit 2), 10 PDXs, and the germline DNA from the patient. These generated a total of 3.58 billion reads (275.6 ± 4.9 million reads per sample) with 3.49 billion (97.49%) mapped to human reference genome hg19/GRCh37 (**S1 Table**). Additionally RNA-seq was performed on all (except the germline) specimen including 10 PDXs and two patient tumor tissue (visit 1 and visit 2) and these produced a total of 2.67 billion reads (221.8 ± 53.3 million reads per sample) with 2.48 billion (92.88%) mapped to the reference genome (**S2 Table**). The deep sequencing depth and high mappability promised comprehensive exploration of the mutational genome and transcriptome landscape.

**Fig 1:**
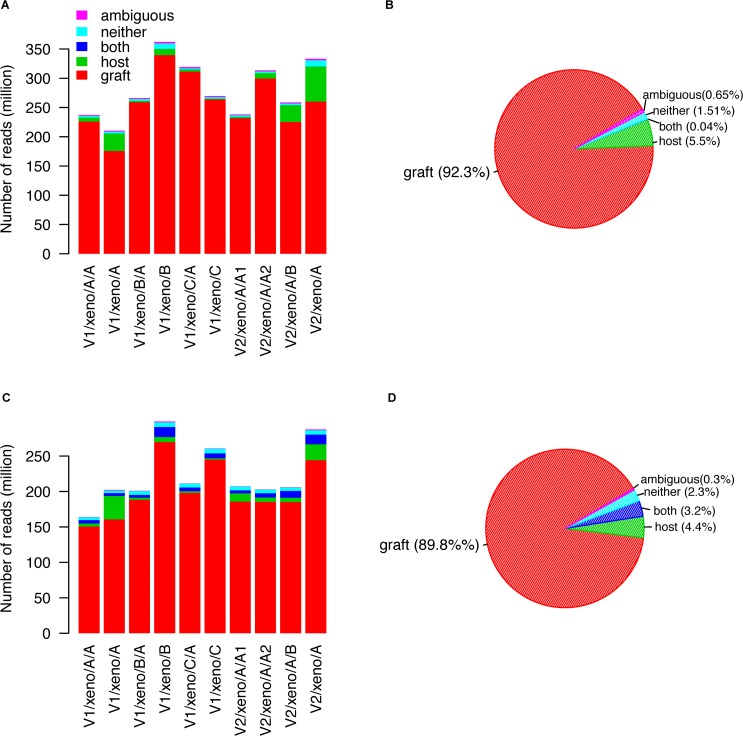
Classification of xenograft whole exome sequencing (panels A and B) and RNA sequencing reads (panels C and D). Reads generated from xenograft samples were divided into five groups including “graft”, “host”, “both”, “neither” and “ambiguous” using tool developed by Conway et al. (A) Reads assignments for 10 xenograft whole exome sequencing data. (B) Average proportion of whole exome sequencing reads assigned to the 5 groups mentioned above. (C) Reads assignments for 10 xenograft RNA-seq data. (D) Average proportion of RNA-seq reads assigned to the 5 groups mentioned above.

We used the classification methods developed by Conway et al [28] to divide all WES and RNA-seq reads into 5 categories including “graft”, “host”, “both”, “neither” and “ambiguous”. Overall, 92.3% and 89.8% of reads were assigned as graft specific for WES and RNA-seq, respectively, demonstrating high species specificity of all xenografts (**Fig 1; S3 & S4 Tables**).

### Somatic mutations

**Fig 2:**
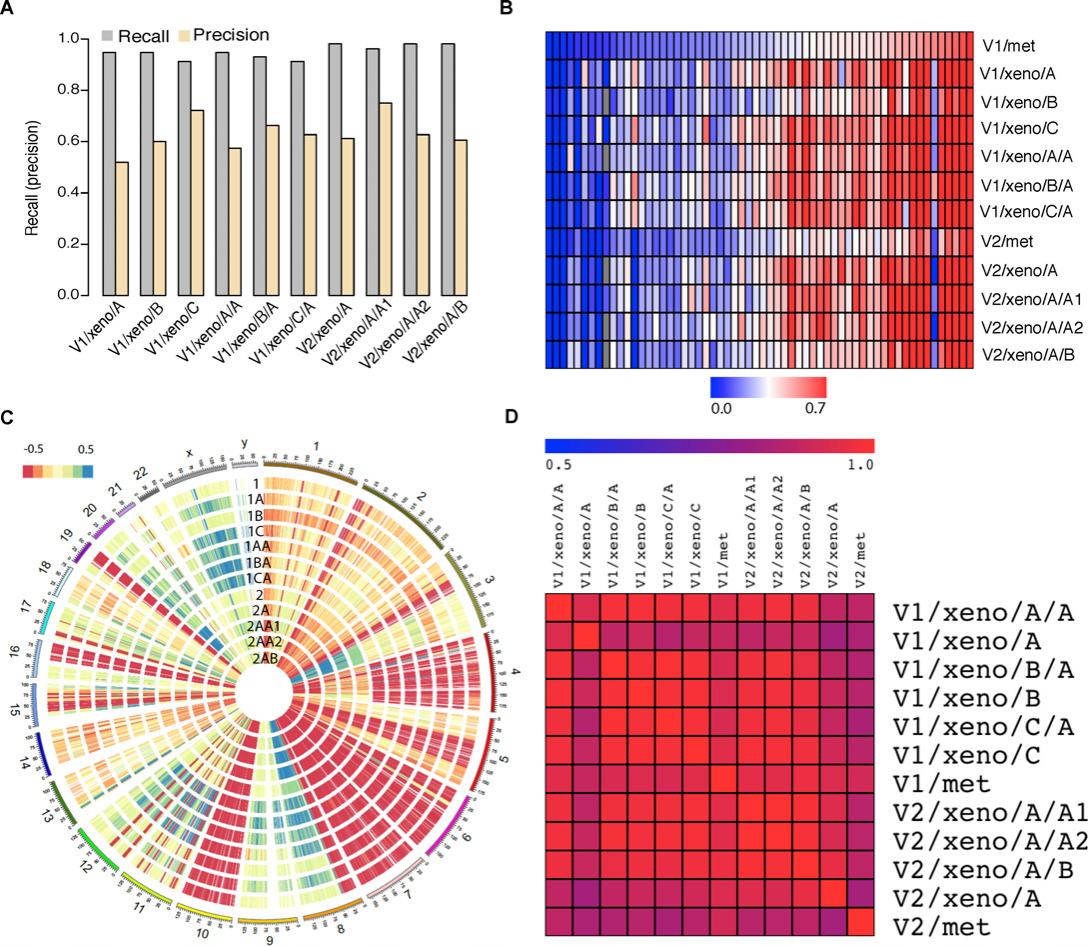
Comparison of genome and transcriptome landscapes between patient tumor tissue and patient derived xenograft models (PDXs). (A) Recall (grey) and precision (tan) of detected somatic mutations. Recall = number of somatic mutations called from both patient tissue and xenograft divided by total somatic mutations called from patient tissue; Precision = number of somatic mutations called from both patient tissue and xenograft divided by total mutations called from xenograft. (B) Heatmap showing the concordance of relative allele frequency of somatic mutations between patient tissues and xenografts. Rows correspond to patients and xenograft samples and columns correspond to 60 selected somatic mutations. (C) Circos plot showing profiles of copy number variation for patient tumor tissues and xenografts. From outside to inside, tracks correspond to 1 = V1/met, 1A = V1/xeno/A, 1B = V1/xeno/B, 1C = V1/xeno/C, 1AA = V1/xeno/A/A, 1BA = V1/xeno/B/A, V1/xeno/C/A, V2/met, V2/xeno/A, V2/xeno/A/A1, V2/xeno/A/A2 and V2/xeno/A/B. (D) Pair-wise gene expression correlation between patient tissues and xenografts. Correlation was measured by Pearson correlation coefficient. Gene expression was measured by log10 (RPKM, Reads Per Kilobase exon per Million mapped reads).

To evaluate genome-wide homogeneity of biopsy tumor tissue and corresponding PDXs, we calculated the recall and precision using all detected somatic mutations. **Fig 2A** shows that most somatic mutations identified from biopsy tissues were rediscovered in PDXs with high recall rate (mean = 0.953, median = 0.958), indicating PDX models faithfully preserved genomic somatic mutations of the original tumors. However, the precision was relatively lower (mean = 0.656, median = 0.657) compared to recall, suggesting there were considerable amount of PDX specific variant calls. This low precision is likely not due to mapping artifacts as the sequence surrounding those variants (101bp centered on the variant) has poor mapping to the mouse genome using BLAT [22]. Presumably, PDX specific mutations mainly originated from two sources: host (mouse) reads contamination and subclone differences. Although we used Xenome to remove host-specific reads, this process is not completely effective given the relatively high similarity between mouse and human genome. Predictably, aligning mouse-derived sequences to the human genome would generate a considerable amount of false positive somatic mutations. In addition, the implantation of tumor cells in NOD-SCID mice could potentially change the frequency of tumor cell populations because of clonal selection, and such subclone drift could also produce many PDX specific mutations. In addition, the relative allele frequencies of somatic mutations were concordant between biopsy tissue and PDXs (**Fig 2B**). For example, on comparing patient tumors and germline DNA a somatic *TP53* mutation (I255F) was detected, which is reported to destabilize the *TP53* protein [29]. This variant was also detected in all PDXs (**S5 Table**). This mutation in *TP53* appeared to be heterozygous in the DNA of the clinical tumor samples and of most PDX’s, but the mutant allele dominated the RNASeq reads in all samples, suggesting the mutant allele specific expression or genome deletion of another copy. After evaluating the CNV data, we found another copy of TP53 was indeed deleted in PDXs with Log_2_R = -1.007 ±0.15. In patient tumor samples, the Log_2_R was -0.6 and -0.65 in metastatic tissue from visit 1 and visit 2, respectively. The lower log_2_R value in patient tissues might due to the lower tumor purity compared to that of PDXs.

DNA tumor purity was estimated at 75% for visit 1 and 76% for visit 2 metastatic samples using the frequency of the most frequent subclone, as computed by PurBayes. The clonality analyses indicated that all three major clones were maintained at relative stable frequencies between patient tissue and PDXs, as well as among different passages of PDXs (**S2 Fig).**

### Copy number variation

Genome wide copy number variation (CNV) analyses revealed high consistence among all samples (**Fig 2C**), with limited lineage-specific CNV differences (e.g. chr3q). We detected somatic copy number gains for *AR* (Log_2_R = 3.91±0.32) and *FGFR1* (log_2_R = 1.49±0.13) genes in tissue biopsies of both visits. As expected, similar levels of copy number gains of *AR* and *FGFR1* were also detected from all PDX samples (**S6 Table**). The *FGFR1* amplification was part of a multi-gene amplified region on chromosome 8 (S3 Fig). The *AR* amplification also included a number of other genes on the high level amplification (3 distal and 1 proximal), as well as two contiguous region of Copy Number gain, with successively diminishing amplitude away from the *AR* gene, potentially indicating that the *AR* amplification occurred as at least three successive events, each of which contributed to an accumulative number of AR gene copies over time. This three level structure was also observed in all xenograft DNA (S4 Fig). The *MLL3* (*KMT2C*) gene was also deleted as part of a 4 gene deleted segment on chromosome 7 from 151253201 to 151884347, which also included *GALNTL5*, *GALNT11*, and *PRKAG2* (S5 Fig). There was also a significant 2.5 Mb deletion on chromosome 15 including *CYP1A1*, *CYP1A2*, *CSK*, *UBE2Q2*, *FBXO22*, *TSPAN3* etc. (S6 Fig). The same two regions were also deleted in the second visit sample. Overall, there were 385 and 451 genes deleted at the Log_2_R ≤ -0.5 level and 97 and 84 genes amplified at the Log_2_R ≥ 0.5 in the first and second visit tumor specimens, respectively.

### Gene fusion

**Fig 3:**
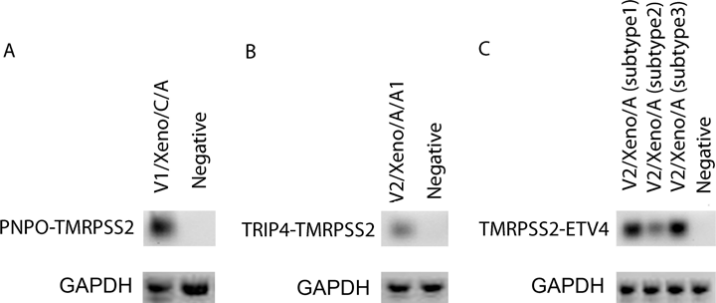
Validation of gene fusion products identified by RNA seq. The upper gel images showed the targeted amplification products, and the lower gel images showed the loading control amplification (GAPDH). (A) The amplification products for PNPO-TMPRSS2 fusion was detected in the xenograft sample V1/xeon/C/A. (B) TRIP4-TMPRSS2 fusion was detected in V2/xeno/A/A1. (C) Three different types of TMRPSS2-ETV4 fusions were detected in V2/Xeno/A. The sample V1/Xeno/A1 was used as negative control.

A total of forty candidate fusions were detected, 8 of which were inter-chromosomal events (**S7 Fig**). We did not detect *TMPRSS2*-*ERG* gene fusion in this patient, however, we found that *TMPRSS2* gene was fused with other partners including *ETV4*, *TRIP4*, and *PNPO* in the visit 1 patient tumor tissue, and all these *TMPRSS2* involved fusion events were rediscovered in PDXs (**S7 Table**). We were able to confirm *TMPRSS2* and *ETV4*, *TRIP4*, and *PNPO* fusion events with QRT-PCR (**Fig 3**). These events were not observed in the visit 2 specimen and the failure to detect these fusions in visit 2 may have been due to RNA degradation and lower tumor content in the biopsy or due to the possibility that clones harboring these fusions represented therapy-sensitive disease.

### Gene expression

Gene expression profiles measured by RNA-seq exhibited an overall high degree of correlation between patient tumor tissue and derived PDX models (Pearson correlation coefficient = 0.933; min: 0.825; max: 0.996) (**Fig 2D**). However, we observed higher expression of *AR* splice variant 7 (*AR*-V7; fold change of 6.25) and *AR* full length (AR-FL; fold change of 4.71) in the first visit metastatic tissue relative to the second visit. The corresponding fold change in the PDXs for ARV7 and AR full length between the first and second visits were 1.60 and 1.29, respectively (**S8 Fig**). We also compared the expression values (measured by RPKM) between visit 1 and visit 2 tumors. When using the threshold of 2 fold change, we detected 461 and 311 genes which were up- and down-regulated in the metastatic tissue from the second visit tumor, respectively (S8 Table). We further performed the pathway analysis using Ingenuity IPA platform, we found that the top enriched pathways for up-regulated genes included *Oxidative Phosphorylation* (*P* = 2.2E-47), *Mitochondrial Dysfunction* (*P* = 1.9E-42) and *Protein Ubiquitination* (*P* = 6.9E-8). The top enriched pathways for down-regulated genes included *Interferon Signaling* (*P* = 6.2E-7), *Clathrin-medicated endocytosis signaling* (*P* = 6.8E-5) and *Activation of IRF by cytosolic pattern recognition receptors* (*P* = 2.6E-4).

## Discussion

The AR inhibitory action of enzalutamide includes competitive blocking of the ligand binding domain of the receptor, inhibition of the translocation of AR to the cell nucleus and inhibiting binding of AR to the DNA [30]. Pre-chemotherapy PSA response rate to enzalutamide (decrease in PSA by 50% of baseline after 12 weeks of treatment) is 78% and a complete or partial soft tissue response rate is 56% [31]. Recently AR-V7 expression has been reported as a molecular predictive factor of enzalutamide efficacy with AR-V7 expression being associated with low PSA response and shorter duration of progression free survival [32]. Our patient did not have a biochemical or a substantive clinical response after 12-weeks of enzalutamide drug exposure and both his metastases as well as the PDX specimens were detected to have AR-V7 expressions. Interestingly minimal decreases in expression levels were observed in the pre to post treatment specimens at 12-weeks. While these observations on AR-V7 expression and resistance to therapy are in agreement with previous reports, the decrease in expression levels of this splice variant post therapy suggests a role for other resistance factors as well including a role for other *AR* splice variants (AR23, AR45) as resistance factors of drug efficacy. Alternately a role for *TMPRSS2*-*ETV4* fusion protein or the expression of other androgen regulated gene fusions with *TMPRSS* interference in determining enzalutamide activity is also unclear. Finally a role for non-AR pathway genes in drug resistance could also be involved. We observed deletion of genes such as *PRKAG2* in tumor tissue and PDXs, a key gene which inactivates enzymes of the fatty acid and cholesterol biosynthesis pathways (a precursor of sex steroids) and which may affect intra tumor sex steroid levels. Increased *FGFR1* copy number was observed in tumor tissue and PDXs. Increase in copy number of this gene has been postulated to play a role in the development of initial hormone resistance to androgen deprivation therapy [33]. Its effect on causing resistance to secondary hormonal therapies such as enzalutamide remains unexplored.

We successfully developed PDXs from mCRPC sites which were able to faithfully represent patient tumor heterogeneity. The concordance in mutation detection and gene expression levels based on exome and RNA-seq data between human tumor and corresponding xenograft tumors was very high. Some of the PDX specific mutations have low accuracy which might be due to mixture of mouse genome. These observations indicate that these PDXs were able to recapitulate the genomic and transcriptomic characteristics of the patient’s underlying cancer. These PDXs now allow the opportunity for evaluating several hypotheses for mechanisms of drug resistance and ultimately develop novel therapeutics that can guide patient intervention. One of the main challenges of developing prostate cancer PDXs for individualized testing of therapies in real-time (i.e. tumor “avatars”) has been a highly variable take-rate, which may be due to several reasons including the source of tissue in prostate cancer (primary vs. metastatic); tissue procurement methods; mouse host strain and engraftment site (subcutaneous or renal sub-capsular). For example, take-rates have been reported to range from 5%–95% for primary prostate cancer, and 25%-95% for metastatic disease [11]. An additional challenge to widespread use of PDX models for lab to bedside translation is the requirement for an immune-deficient host. As a result, PDX models are unable to faithfully recapitulate the relationships between different cell types and cytokines in the tumor microenvironment. This is of particular relevance when evaluating cancer immunotherapies or the role of the immune system in disease progression and/or response to AR-targeted therapy or chemotherapy. Another challenge for the successful adoption of using PDXs to enhance precision medicine is the feasibility of translating results from the lab to the bedside in real time after obtaining a functional validation of targetable genes. Finally, the availability of drugs that can be used against the driver mutations identified in this and other studies [4,34] is another practical consideration

## Conclusions

PDX models remain an attractive option as functional tumor models if they are able to reflect the tumor heterogeneity observed in the original patient tumor through initial stages of passaging [35]. We successfully elucidated the establishment of sequential PDX models from a patient on enzalutamide therapy in castrate resistant stage. Attempts to evaluate the underlying drug resistance mechanisms using this model system for the delivery of precision medicine in future are at present on-going.

## Supporting Information

S1 Fig Schematic representation of relationship between patient tissue (visit1 and visit2) and derived xenograft models (1st and 2nd generation). (TIFF)

S2 Fig The output of PurBayes is visualized by plotting the mutant allele counts versus the total reads at each site for V1/Met and derived PDXs (A-G), V2/Met and derived PDXs (H-L). Three clonal populations were indicated as black, blue and green lines, respectively. The corresponding dotted lines indicate the 95% confidence interval.
(TIFF)

S3 Fig Screenshot of UCSC genome browser showing multi-gene amplified region on chromosome 8 harboring gene *FGFR1*. Y-axis indicates the log2 ratio of reads coverage between tumor and germline DNA. V1/Met and derived PDXs were indicated as red tracks, V2/Met and derived PDXs were indicated as green tracks.
(TIFF)

S4 Fig Screenshot of UCSC genome browser showing multiple levels of copy number gain around the AR gene (highlighted) on chromosome X. Y-axis indicates the log2 ratio of reads coverage between tumor and germline DNA. V1/Met and derived PDXs were indicated using red tracks, V2/Met and derived PDXs were indicated using green tracks.
(TIF)

S5 Fig Screenshot of UCSC genome browser showing 4 gene deleted segment on chromosome 7. Y-axis indicates the log2 ratio of reads coverage between tumor and germline DNA. V1/Met and derived PDXs were indicated as red tracks, V2/Met and derived PDXs were indicated as green tracks.
(TIFF)

S6 Fig Screenshot of UCSC genome browser showing 2.5 Mb segment deletion on chromosome 15. Y-axis indicates the log2 ratio of reads coverage between tumor and germline DNA. V1/Met and derived PDXs were indicated as red tracks, V2/Met and derived PDXs were indicated as green tracks.
(TIFF)

S7 Fig Circos plot showing 40 candidate gene fusions detected from patient tissue and xenografts. TMPRSS2 encompassing fusions were indicated by red links.
(TIFF)

S8 Fig Isoform specific expression for AR variant 7 (ARV7, left) and AR full length (ARF, right). SRPM (splice reads per million) is number of splice reads that specifically support ARV7 (or ARF) normalized by total splice reads.
(TIFF)

S1 Table Whole exome sequencing (WES) statistics for 13 samples including 10 patient derived xenografts, patient tumor (Visit1 and Visit2) and blood tissue (Visit1). (XLSX)

S2 Table RNA sequencing (RNAseq) statistics for 12 samples including 10 patient derived xenografts, patient tumor (Visit1 and Visit2). (XLSX)

S3 Table Whole exome sequencing reads classification of 10 patient derived xenografts. (XLSX)

S4 Table RNA-seq reads classification of 10 patient derived xenografts. (XLSX)

S5 Table Clinically relevant DNA somatic variants. Reads supporting reference allele and mutant allele are shown for both the WES and RNAseq data. Gene name (codon change) are provided.
(XLSX)

S6 Table Copy number variation (CNV) in clinically relevant genes. (XLSX)

S7 Table TMPRSS2 encompassed fusions. Number of paired-end reads supporting the fusion.
(XLSX)

S8 Table Differentially expressed genes (DEG) between patient tumor tissue before and after treatment (i.e. Visit 1 vs Visit 2). DEGs were defined as those genes with RPKM fold change > 2 or < 0.5.
(XLSX)

S1 Animal Methods Description of mice anesthesia and Euthanasia methods. (DOCX)
