# Supplementary figures and images for: Mutational Landscapes of Sequential Prostate Metastases and Matched Patient Derived Xenografts during Enzalutamide Therapy
Source: PLoS One. 2015 Dec 22;10(12):e0145176. doi: 10.1371/journal.pone.0145176 (PMC4687867; doi:10.1371/journal.pone.0145176)

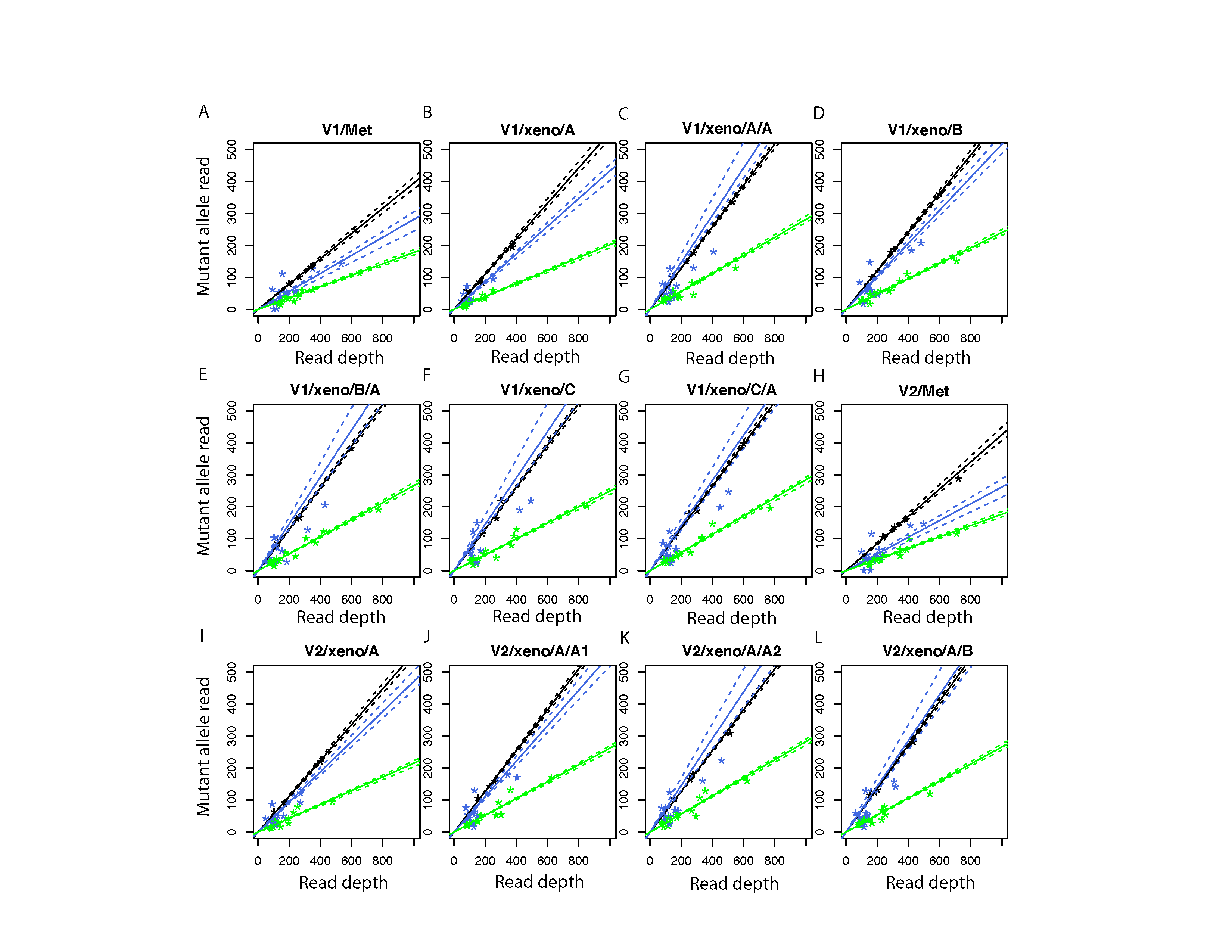

Supplement: S2 Fig — Three clonal populations were indicated as black, blue and green lines, respectively. The corresponding dotted lines indicate the 95% confidence interval. (TIFF) [file pone.0145176.s002.tiff]

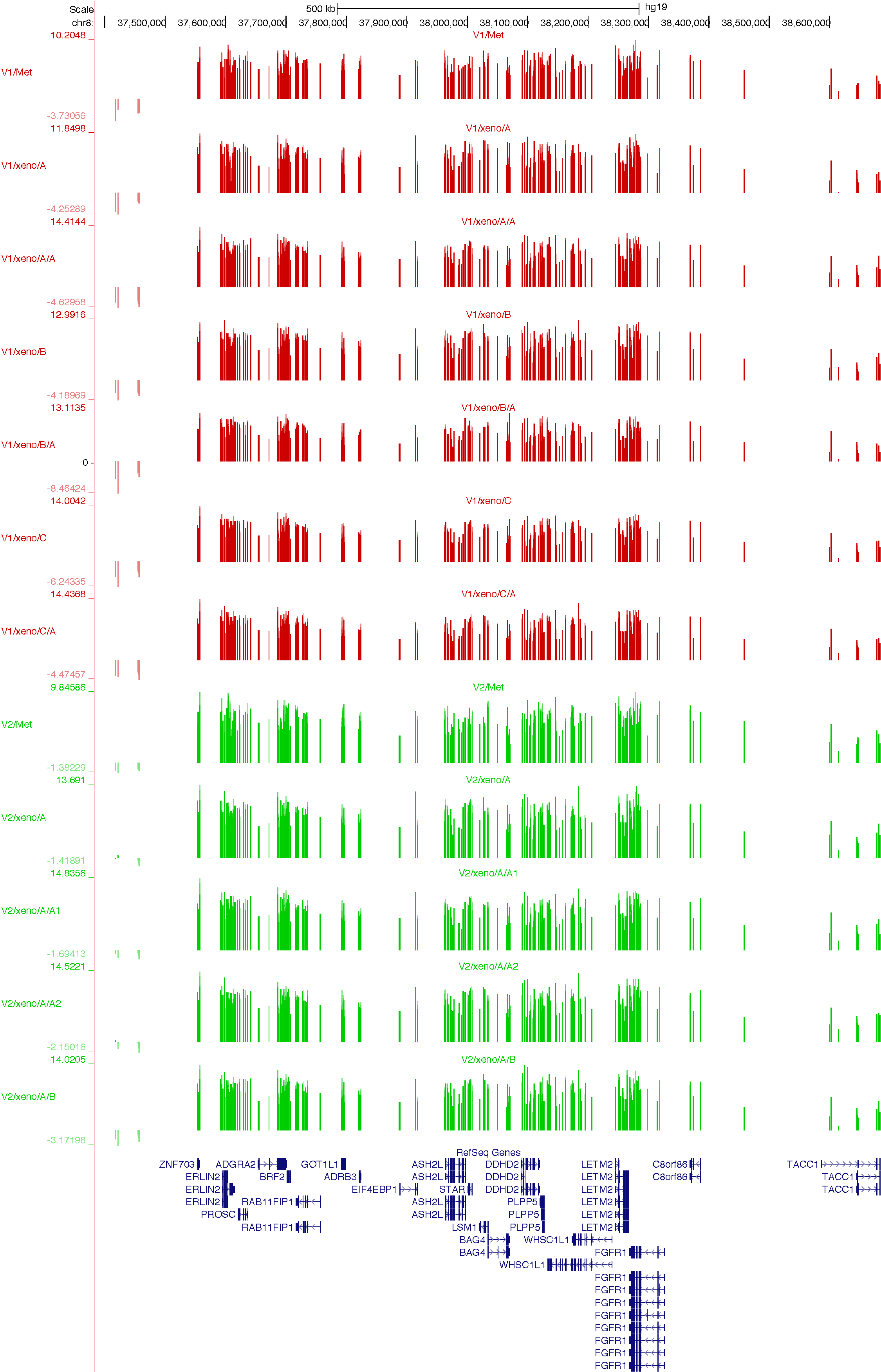

Supplement: S3 Fig — Y-axis indicates the log2 ratio of reads coverage between tumor and germline DNA. V1/Met and derived PDXs were indicated as red tracks, V2/Met and derived PDXs were indicated as green tracks. (TIFF) [file pone.0145176.s003.tiff]

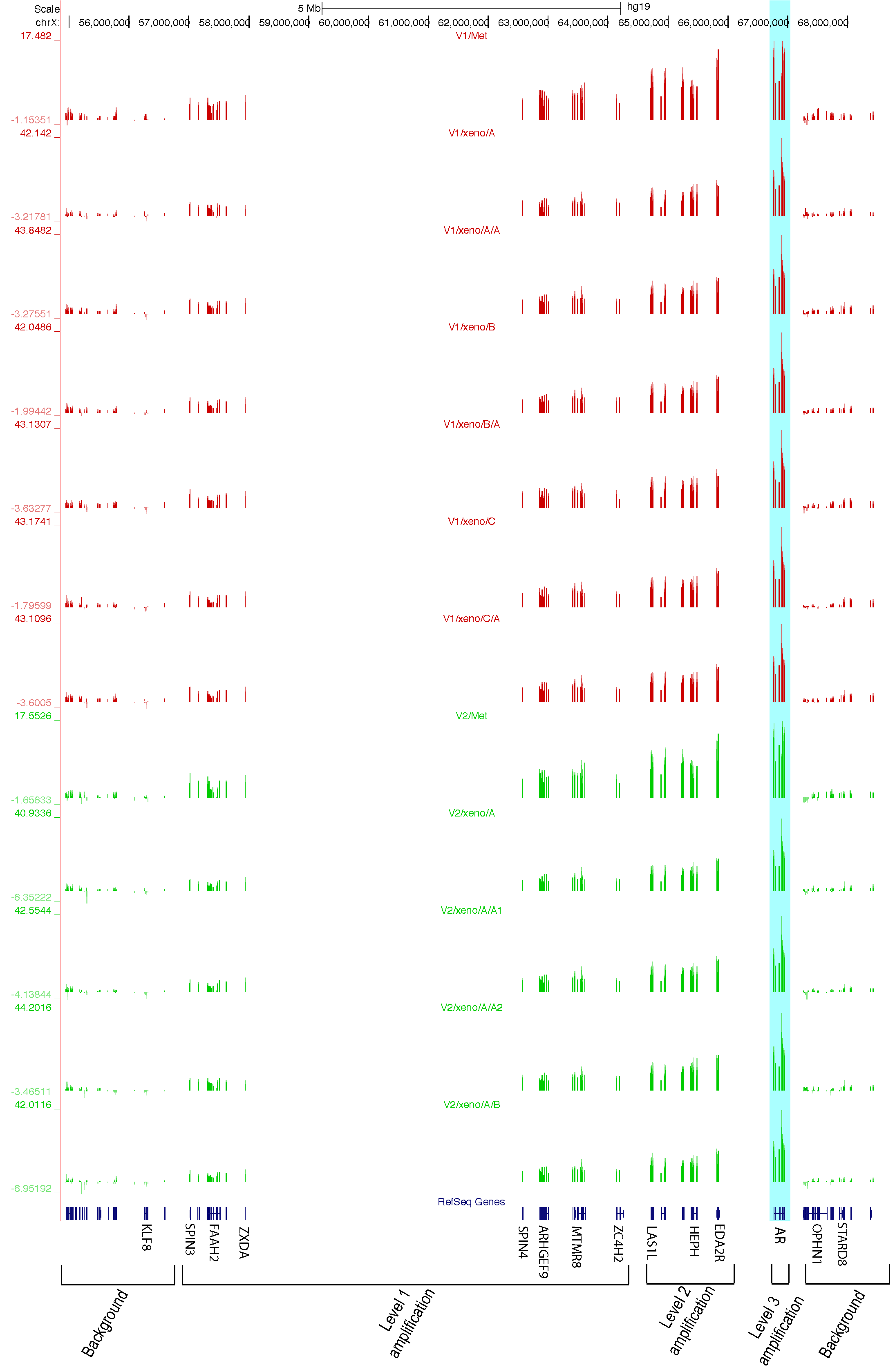

Supplement: S4 Fig — Y-axis indicates the log2 ratio of reads coverage between tumor and germline DNA. V1/Met and derived PDXs were indicated using red tracks, V2/Met and derived PDXs were indicated using green tracks. (TIF) [file pone.0145176.s004.tif]

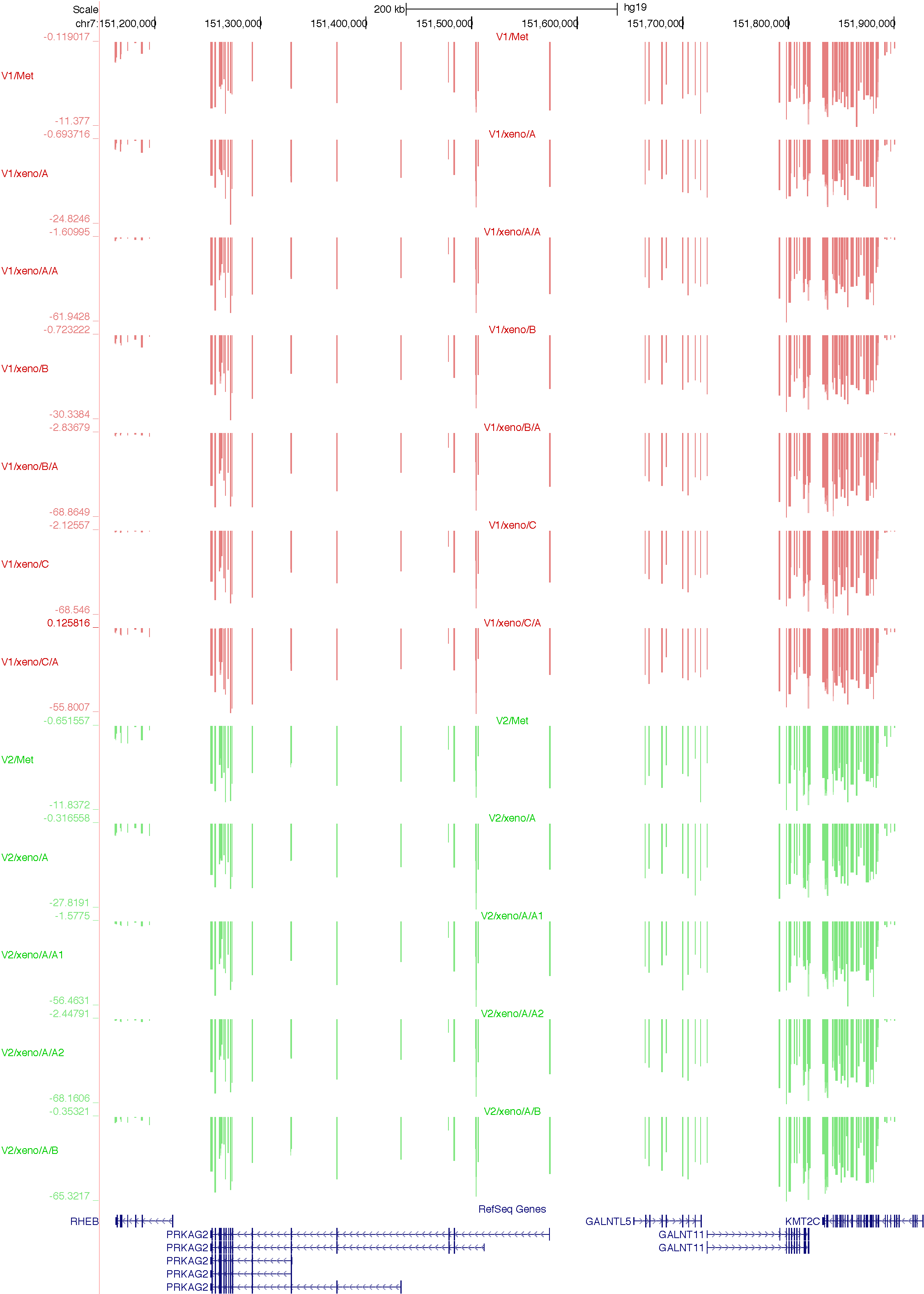

Supplement: S5 Fig — Y-axis indicates the log2 ratio of reads coverage between tumor and germline DNA. V1/Met and derived PDXs were indicated as red tracks, V2/Met and derived PDXs were indicated as green tracks. (TIFF) [file pone.0145176.s005.tiff]

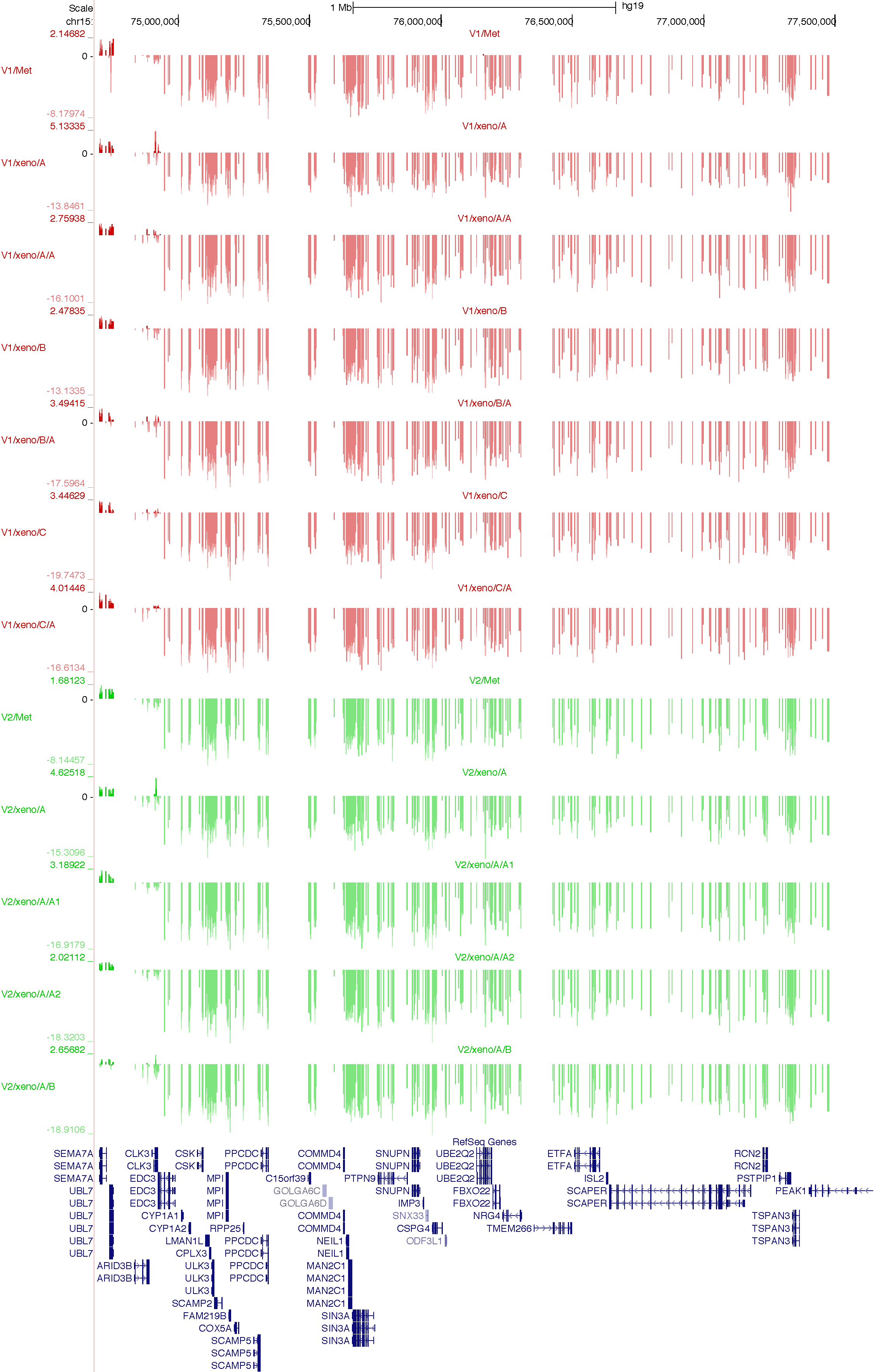

Supplement: S6 Fig — Y-axis indicates the log2 ratio of reads coverage between tumor and germline DNA. V1/Met and derived PDXs were indicated as red tracks, V2/Met and derived PDXs were indicated as green tracks. (TIFF) [file pone.0145176.s006.tiff]

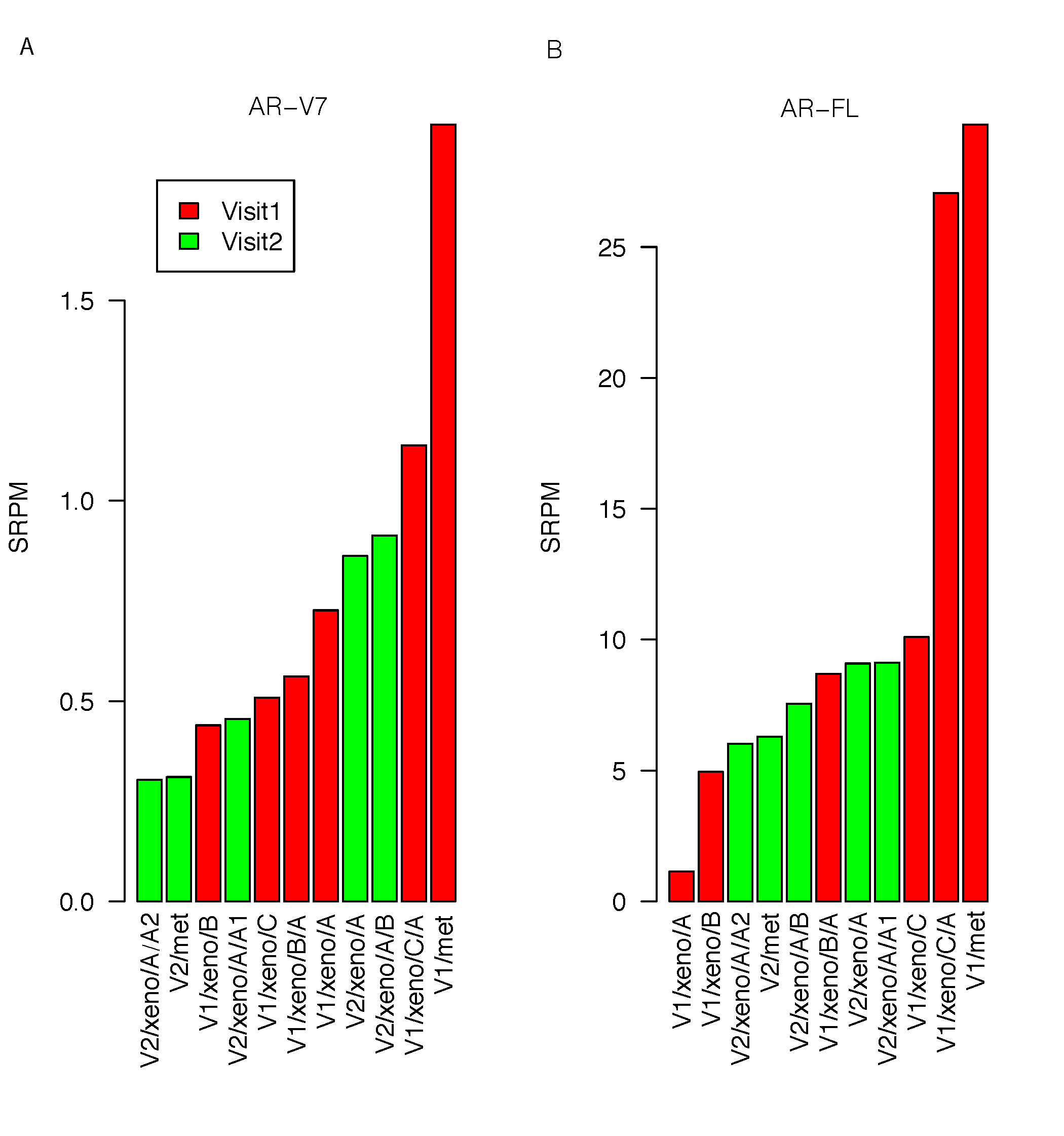

Supplement: S8 Fig — SRPM (splice reads per million) is number of splice reads that specifically support ARV7 (or ARF) normalized by total splice reads. (TIFF) [file pone.0145176.s008.tiff]
