# Supplementary material for: Mutational Landscapes of Sequential Prostate Metastases and Matched Patient Derived Xenografts during Enzalutamide Therapy
Source: PLoS One. 2015 Dec 22;10(12):e0145176. doi: 10.1371/journal.pone.0145176 (PMC4687867; doi:10.1371/journal.pone.0145176)
Supplement: S1 Animal Methods — (DOCX) [file pone.0145176.s017.docx]

**Supplementary PDX Methods**

Methods for animal euthanasia

Anesthesia method:

**INDUCTION:**

| **Agent** | **Dose(mg/kg)** | **Route** | **Length of time anesthesia session will last** |
| --- | --- | --- | --- |
| ketamine ( under the renal capsule implantation) | 100 | ip | 10 mins |
| xylazine (under the renal capsule implantation) | 10 | ip | 10mins |

**MAINTENANCE:**

| **Agent** | **Dose(mg/kg)** | **Route** | **Frequency of administration** | **Assess depth of anesthesia** |
| --- | --- | --- | --- | --- |
| ketamine | 33mg/kg | ip | as needed until the surgery is finished (20 minutes) | toe pinch |
| xylazine | 3.3mg/kg | ip | as needed until the surgery is finished (20 minutes) | toe pinch |

Euthanasia method:

Euthanasia by CO2 inhalation as recommended by the Mayo Clinic Department of Comparative Medicine.

The age of mice: 4-6 weeks

Size: ~20g per mouse

Gender: Male
